# Video-assisted self-reflection of resuscitations for resident education and improvement of leadership skills: A pilot study

**DOI:** 10.1007/s40037-021-00690-9

**Published:** 2021-11-16

**Authors:** Lauren Kava, Kerin Jones, Robert Ehrman, Laura Smylie, Matthew McRae, Elizebeth Dubey, Brian Reed, Anne Messman

**Affiliations:** 1 Lincoln Hospital, New York City, USA; 2 Detroit Receiving Hospital, Detroit, MI USA; 3 Sinai-Grace Hospital, Detroit, MI USA; 4 Covenant Hospital, Saginaw, MI USA

**Keywords:** Resuscitation, Medical education, Leadership, Resident education, Video analysis

## Abstract

**Introduction:**

One of the most challenging aspects of Emergency Medicine (EM) residency is mastering the leadership skills required during a resuscitation. Use of resuscitation video recording for debriefing is gaining popularity in graduate medical education. However, there are limited studies of how video technology can be used to improve leadership skills in the emergency department. We aim to evaluate the utility of video-assisted self-reflection, compared with self-reflection alone, in the setting of resuscitation leadership.

**Methods:**

This was a prospective, randomized, controlled pilot study conducted in 2018 at an urban level 1 trauma center with a three-year EM residency program. The trial included postgraduate year (PGY) 2 and 3 residents (*n* = 10). Each resident acted as an individual team leader for a live real-time resuscitation in the emergency department. The authors classified a patient as a resuscitation if there was an immediate life- or limb-threatening disease process or an abnormal vital sign with an indication of hypoperfusion. Each resident was recorded as the team leader twice. Both control and intervention groups produced written self-reflection after their first recording. The intervention group viewed their resuscitation recording while completing the written reflection. After their reflection, all participants were recorded for a second resuscitation. Two faculty experts, blinded to the study, scored each video using the Concise Assessment of Leader Management (CALM) scale to measure the leadership skills of the resident team leader.

**Results:**

Five PGY‑3 and five PGY‑2 residents participated. The weighted kappa between the two experts was 0.45 (CI 0.34–0.56, *p* < 0.0001). The median gain score in the control group was −1.5 (IQR) versus 0.5 in the intervention group (IQR).

**Discussion:**

Video-assisted self-reflection showed positive gain score trends in leadership evaluation for residents during a resuscitation compared with the non-video assisted control group. This tool would be beneficial to implement in EM residency.

**Supplementary Information:**

The online version of this article (10.1007/s40037-021-00690-9) contains supplementary material, which is available to authorized users.

## Introduction

Leadership failure can have disastrous results. A report by The Joint Commission found that team leadership failures in acute healthcare settings were associated with more than 50% of adverse events [1]. Exacerbating this is that medical education has been slow to incorporate explicit leadership training into graduate medical education [2, 3]. Many residents develop leadership skills during patient care through trial and error, observation, feedback, and reflection [4]. Specific to Emergency Medicine (EM), the modality by which team leadership is effectively assessed has been elusive [4, 5].

One of the most challenging aspects of EM residency training is mastering the leadership skills required during a resuscitation[4]. During a resuscitation, the team leader is tasked with the responsibility of gathering information from multiple sources, formulating a differential diagnosis, initiating interventions, and communicating clearly and professionally with the patient and team members. These tasks must be performed quickly and competently in critically ill patients. There are critical moments where the leader’s actions, communication and decisions have a large impact on the patient. This is usually an intense period during which leaders can be cognitively overloaded, making it inappropriate or impossible for them to reflect on their leadership abilities in real time [6].

Simulation allows for complete control of the resuscitation case to practice logistics, understand procedures, and demonstrate learning points. However, the disadvantage of simulated cases is that they lack the authenticity, pressure and investment that occurs when treating a real, live unstable patient [7, 8].

Debriefing and self-reflection are ways to address the strengths and weaknesses of leadership skills. This practice of analyzing decisions that unfold quickly and have a high impact is commonly used in elite athletics. The exercise takes advantage of behaviorism and visualization to understand what they did, why they did it, and how they will change their behavior next time for a better outcome. Additionally, athletic reflection is often video-assisted. It is seen in the stereotypical situation of football players watching films before a game and has been studied in volleyball and tennis, showing improved long-term memory in tactical knowledge, improved strategy planning, and more meaningful awareness of the environment [9, 10].

There is a tremendous opportunity to translate video-assisted self-reflection to leadership skills in resuscitations. Video-assisted feedback has been shown to improve algorithmic compliance in Advanced Trauma Life Support (ATLS) and cardiopulmonary resuscitation (CPR), with the premise that objective evidence of performance improves motivation and self-efficacy [11, 12].

The basis for the hypothesis that skills will improve more amongst residents who are able to watch videos of themselves leading a resuscitation has its roots in cognitivism. Reflection is critical in cognitivism, the first step of which is to return to and replay the learner’s experience [13]. This step is facilitated by providing learners with a video of themselves as team leader so that the learner can replay, with 100% accuracy, the events of the resuscitation. In cognitivism, the onus of learning is firmly on the learner, with the teacher acting as a guide to help the learner “learn how to learn [13]”.

In this study, we assess the utility of resuscitation video-assisted self-reflection compared with self-reflection alone.

## Methods

We conducted a prospective, randomized, controlled pilot study. Audio and video were recorded of EM residents functioning as team leader during a resuscitation in real-time patients presenting to the emergency department. Recordings were uploaded to an encrypted web-based storage system (Wayne State One Drive) accessible only to the study authors. The Wayne State University School of Medicine Institutional Review Board classified this study as program evaluation/quality improvement and granted exemption. Patient consent was not required based on this classification as quality improvement. We have also adhered to the CONSORT guideline [14].

### Setting and population

This study was conducted at an urban, level 1 trauma center with approximately 90,000 adult visits per year and a three-year EM residency program. A total of ten PGY‑2 and PGY‑3 EM resident physicians volunteered to enroll in the study. Recruitment consisted of an email sent to the second- and third-year classes by a PGY‑3 participating in the study offering the opportunity to participate. Study staff would then be present in the emergency department during their shifts to capture data. Inclusion criteria for resident participation included (1) second- or third-year EM residents in good standing, (2) on an EM rotation during the months of August, September, and October 2018, and (3) having a resuscitation on a day that a member of the study staff was present to record. Patients were included if they were triaged as a resuscitation by emergency medical services or a triage nurse, which comprises patients who have an immediate life- or limb-threatening disease process, or patients with a vital sign abnormality with clear evidence of hypoperfusion.

### Study protocol

The ten resident physicians were randomized into two groups, with an allocation ratio of 1:1. The control group consisted of three PGY‑2 residents and two PGY‑3 residents. The intervention group consisted of two PGY‑2 residents and three PGY‑3 residents. Participants in both groups were recorded leading an adult resuscitation twice for a total of 20 recordings. After the first resuscitation, all ten residents participated in guided self-reflection regarding their capabilities as team leader. For all ten residents, the self-reflection was guided in that they were provided with a “Resident Reflection Sheet” (Supplemental File 1 of the Electronic Supplemental Material) as opposed to reflecting on their capabilities in an unstructured way. The intervention group watched a recording of their resuscitation while they completed the “Resident Reflection Sheet,” whereas the control group completed the “Resident Reflection Sheet” without access to their recording. All residents in each group were then recorded leading a second resuscitation. No feedback or discussion regarding the resident’s self-reflection was provided prior to completion of the second resuscitation.

Two faculty experts with experience in residency leadership and education reviewed all 20 videos to assess the leadership abilities of the resident functioning as the team leader during the case. The faculty experts reviewed the videos in random order and did not know whether they were viewing a video from the control or the intervention group, nor were they aware whether they were viewing a video from the first group of resuscitation cases (prior to the guided self-reflection) or after the guided self-reflection. The faculty experts scored all videos using the Concise Assessment of Leader Management (CALM) instrument (Supplemental File 2 of the Electronic Supplementary Material), a previously validated instrument designed to require minimal user training to assess leadership skills and to provide formative feedback to learners [15]. Rater training of the faculty experts consisted of a consensus meeting to agree on interpretation of assessment variables, and three practice cases wherein the experts rated cases independently and discussed differences in scoring between the two. These practice cases were not included in the overall cohort. Residents were unaware that their performance would be evaluated by the CALM instrument.

The key outcome measure in this study was the CALM score before and after video-assisted reflection.

### Data analysis

Descriptive statistics were used to explore resident characteristics and evaluation timing; ordinal data were described using medians and interquartile ranges (IQRs). Individual scores for each resident generated using the CALM instrument for the pre- and post-test assessments were summed for a total score. A gain score was calculated for each resident by subtracting pre-test scores from post-test scores. Total gain scores on the CALM instrument between the control and intervention groups were compared. Gain scores between the control and intervention group for the CALM instrument were reported as medians (IQR) with a 95% confidence interval (CI). Agreement between the two faculty experts was assessed using the weighted kappa statistic. Consensus was also assessed between the two faculty experts, and was defined as a variation of no greater than one point on the CALM score for each of the 15 individual items in the instrument to account for subjectivity in ratings while still allowing for identification of significant differences in ratings.

## Results

Five PGY‑2 residents and five PGY‑3 residents participated in the study out of 28 possible candidates. All video recording took place in September and October 2018. The median time between the first resuscitation and resident reflection was three days (mean 9.2 days). The median time between the first and second resuscitation recordings was 16.5 days (mean 19.3 days). Resuscitation cases encountered during the study period are described in Tab. 1.

**Table 1:** Descriptions of the resuscitation cases per resident divided between pre- and post-reflection, as well as delineation between control (written reflection of first resuscitation) and intervention (video-assisted written reflection of first resuscitation) group with corresponding post-graduate year (PGY)

	Pre-reflection case	Post-reflection case	PGY
**Control**			
*Resident 1*	Altered mental status with respiratory distress (alcohol withdrawal)	Sudden onset abdominal pain in pregnancy with hypotension	3
*Resident 2*	Respiratory distress (pulmonary edema)	Chest pain with abnormal electrocardiogram	2
*Resident 3*	Respiratory distress (pulmonary edema)	Respiratory distress (bronchospastic)	2
*Resident 4*	Tachyarrhythmia	Respiratory distress (pulmonary edema)	2
*Resident 5*	Syncope with respiratory distress	Altered mental status with respiratory failure (unknown etiology)	3
**Intervention**			
*Resident 6*	Respiratory distress (pulmonary edema)	Altered mental status with respiratory failure (opioid overdose)	3
*Resident 7*	Respiratory distress (bronchospastic)	Tachyarrhythmia	3
*Resident 8*	Respiratory distress (bronchospastic)	Status epilepticus	2
*Resident 9*	Hypertensive encephalopathy	Altered mental status (seizure)	3
*Resident 10*	Hypotension with syncope	Cardiac arrest	2

Consensus was achieved between the two faculty experts for 95% of the 300 total variables on the CALM assessment tool (15 variables for each video times 20 videos). The weighted Kappa between the two experts was 0.45 (CI 0.34–0.56, *p* < 0.0001).

Tab. 2 shows the total median composite gain scores of control vs. intervention, the median gain scores generated by the expert reviewers between the residents’ first and second recordings, and also describes all 15 items assessed with the CALM instrument. The median composite gain score in the control group was −1.5 (IQR) versus 0.5 in the intervention group (IQR). The 95% CI for the difference in the medians between the groups was −8.5 to 4.0. Gain scores for the individual faculty experts were also compared and no significant difference was found for either the control or intervention group (data not shown).

**Table 2:** Median composite gain scores between control and intervention. 95% CI for the difference between the two medians is −8.5 to 4.0 by Hodges-Lehman estimation

Assessment	Control Median (Q1, Q3)	Intervention Median (Q1, Q3)
Clear role as leader	0 (0, 0)	−1 (−2, 0)
Style appropriate	0 (−1, 0)	−1 (−1, 0)
Voice appropriately loud and clear	0 (−1, 0)	0 (−2, 1)
Addresses people explicitly	1 (0, 2)	−1 (−1, 0)
Reinforces closed loop communication	−1 (−1, 1)	0 (−1, 1)
Acknowledges roles	0 (0, 1)	0 (0, 1)
Directs team effectively	−1 (−1, 0)	0 (−1, 0)
Balances work load of team	0 (0, 1)	−1 (−2, 1)
Engages team members in decision making	0 (0, 1)	1 (0, 2)
Summarizes case periodically	−1 (−1, −1)	0 (0, 1)
Prioritizes task order	−1 (−2, −1)	1 (0, 1)
Maintains global view	0 (−1, 0)	0 (0, 1)
Periodically reassesses patient	−1 (−1, 0)	2 (1, 2)
States next steps in patient care	−1 (−1, 0)	1 (0, 2)
Aware of limitations	0 (0, 0)	0 (0, 0)
*Composite score*	−1.5 (−2, 0.5)	0.5 (−3.5, 4.50)

## Discussion

Functioning effectively as the team leader during a resuscitation is a difficult task to master [12, 16]. Resident learners may not be able to identify their shortcomings as team leader given that physicians have been found to be poor at self-assessment [17]. Prior work has shown that video analysis leads to improved algorithmic compliance in trauma resuscitation cases and in cardiopulmonary resuscitation [11, 12, 18, 19]. We have applied this line of thinking to leadership in resuscitations, where there is not a specific algorithm to comply with, to test whether residents watching videos of themselves leading a resuscitation will gain the same benefit conferred to physicians who are able to watch themselves following the ATLS or CPR algorithm.

This pilot study found a positive trend in gain scores using the CALM instrument and demonstrates a positive effect of incorporating video feedback into leadership training for EM residents. Previous studies have examined the relationship between resident self-assessment and faculty assessment of resident performance when watching videos of residents performing elective laparoscopic surgery and in placing ultrasound-guided internal jugular triple lumen catheters and found no correlation between the residents’ self-assessment scores and the scores given by faculty after watching the videos [20, 21]. This study differs in that we were not looking for a correlation between these two scores but rather for an improvement in objective faculty assessment of resident performance if the residents were provided with their videos to watch during their guided self-assessment.

Given aspects of the CALM instrument such as prioritizing task order or stating next steps in patient care are likely obvious to learners when watching themselves, it may not be surprising that gain scores showed improvement in the intervention group. However, it is important to note that the resident had no knowledge of being assessed by the CALM instrument and therefore did not know which specific domains were to be judged. Clearly for the purpose of this study, we could not provide the resident with the CALM assessment tool in order to not confound our results, but we expect the effects of video feedback to be significantly magnified once the residents are aware of its purpose. Additionally, residents can be trained to appraise their videos critically so that they can obtain maximum benefit from watching themselves and can target many different skills, in addition to leadership.

This project’s assessment of leadership skills provides benefit of data for several competencies of the Emergency Medicine Milestones Project [22]. The Accreditation Council of Graduate Medical Education (ACGME) and the American Board of Emergency Medicine created the Emergency Medicine Milestones Project in 2012 to provide competency-based assessment recommendations for EM residents in ACGME-approved residencies. Specifically, the Patient Safety competency (SBP1) requires that an EM resident “identifies situations when the breakdown in teamwork or communication may contribute to medical error” in order to achieve a level four milestone. The Practice-based Performance Improvement competency (PBL1) requires that a resident “performs self-assessment to identify areas for continued self-improvement” to achieve a level three milestone. Finally, the Team Management competency (ICS2) generally requires that a resident “leads patient-centered care teams, ensuring effective communication and mutual respect among members of the team” in order to achieve any level of this milestone and thus is the competency most well addressed with video analysis. All of these milestones can be objectively assessed with the methods employed in this study and may be of value to residency programs wishing to identify further objective data for these competencies [22].

Another potential usage of video recording for feedback in EM is in the realm of directed coaching. This has already been studied in general surgery, wherein resident physicians were video recorded performing a surgical procedure. Each resident then met with a supervising attending physician for 1 hour and were provided with targeted feedback while watching the video together. This resulted in improved and individualized teaching and feedback [23]. Soucisse et al. found improved surgical skills in general surgery residents filmed performing side-by-side intestinal anastomoses who were then provided coaching by faculty while reviewing the video together [24]. The usage of video recordings with debriefing and coaching by faculty represents a future direction for using video recording in EM residency training that merits exploration. Additional recording modalities, such as usage of a head-mounted GoPro, have been studied in general surgery residents with results demonstrating feasibility of this modality and utility in providing more meaningful feedback [25]. Use of an inexpensive, easy-to-acquire recording device such as a GoPro may improve the feasibility of video recording and feedback in EM.

This study begins to shed light on the difficulties of effective leadership development during resuscitations and the benefit of reflection with objective video recording. Our goal would be to incorporate this exercise in residency education. Other than the initial cost of the recording equipment, this is not a practice that requires continuous funding or significant faculty time or oversight. Additionally, residents can perform this activity on their own and present their findings to faculty or program leadership, which is a major advantage over many other curricular innovations.

### Limitations

The primary limitations are the small sample size and single site nature of this pilot study. The study participants were also drawn from a convenience sampling of EM residents, which may confound the results. There was also some variability in the timing of when participants completed their self-reflection relative to completion of the resuscitation case, which may affect the influence of the self-reflection exercise. Given that the resuscitations were on live patients presenting to the emergency department rather than simulated cases, there was heterogeneity in the complexity of the case presentations which could affect the resident’s performance. Additionally, reflection was done solely by the resident and not with the whole medical team (nursing staff, respiratory therapist, pharmacy staff), which may give further insight into the residents leadership abilities.

## Conclusions

Our study showed a positive trend in gain score evaluation of leadership skills for residents utilizing video-assisted self-reflection after resuscitation compared with the non-video-assisted control group. These initial results are very encouraging and support further exploration with a larger sample size and multiple sites.

## Supplementary Information

1. Resident Reflection Sheet: *used by each resident to guide self reflection after each resuscitation. The control group only used the resident reflection sheet. The intervention group filled out the resident reflection sheet while watching their video.*

2. Concise Assessment of Leader Management: *Previously validated instrument designed by Nadkarni LD, et. al to require minimal user training to assess leadership skills and provide formative feedback. Used by our attending experts to score all 20 videos.*
