# Supplementary material for: Video-assisted self-reflection of resuscitations for resident education and improvement of leadership skills: A pilot study
Source: Perspect Med Educ. 2021 Nov 16;11(2):80–5. doi: 10.1007/s40037-021-00690-9 (PMC8940988; doi:10.1007/s40037-021-00690-9)
Supplement: Supplementary file 1 — 1. Resident Reflection Sheet: used by each resident to guide self reflection after each resuscitation. The control group only used the resident reflection sheet. The intervention group filled out the resident reflection sheet while watching their video. [file 40037_2021_690_MOESM1_ESM.pdf]

Resident Reflection Sheet

Intended Use: The intended use of the "Resident Reflection Sheet" is to guide the resident in assessing their technical and non-technical skills as a code leader in a medical resuscitation code. The intervention group will fill this out as they watch themselves lead the medical resuscitation. The control group will fill this out without watching the resuscitation. This tool is adapted from Dr. Fernandez's tool "EM Resuscitation Team Leadership Measurement, " and Dr. Kessler's tool "Concise Assessment of Leadership Management." However, our use is intended to help guide the resident in reflection of how they executed a medical resuscitation.

Goals of Tool: The goal of the "Resident Reflection Sheet" is to help the resident acknowledge skills they excel at and where they require improvement. The idea is that if you can watch yourself and reflect, then you can become more self-aware, to increase confidence in strengths, and help you acknowledge weaknesses. The actual responses of where the resident classifies herself, is less important than helping the resident develop some type of opinion on their own performance.

Interpretation of Answers:

Answer:	Interpretation:
□ N/A	*Unable to answer. This component was not involved in our resuscitation.*
□ Needs Improvement	*I was unable to perform this skill. The attending had to step in and take over, or that skill or task was just not accomplished*
□ Adequate	*I accomplished the task, or displayed the skill. I had significant difficulty or needed significant support or verbal direction from the attending.*
□ Good	*I felt comfortable with performing the task or skill. I required intermittent guidance from attending, and relied on the attending for support.*
□ Mastered	*I feel extremely confident in this skill or task. I perform it as if I were an attending physician. I need no support.*

Components of History	
Did I obtain and understand the full history of present illness?	☐ N/A ☐ Needs Improvement ☐ Adequate ☐ Good ☐ Mastered
Did I obtain a full support history (*PMH / PSH / Meds / Allergies / FH / social history*)?	☐ N/A ☐ Needs Improvement ☐ Adequate ☐ Good ☐ Mastered
Did I use available resources (*EMS / EMR / Family*) supplement story or when patient wasn't able to give history?	☐ N/A ☐ Needs Improvement ☐ Adequate ☐ Good ☐ Mastered
Did I have a good balance of time between history and intervention? (*example, did I continue to ask patient questions when they obviously needed emergent intubation?*)	☐ N/A ☐ Needs Improvement ☐ Adequate ☐ Good ☐ Mastered
****THIS IS WHAT I PLAN TO DO DURING MY NEXT CODE TO IMPROVE MY HISTORY:**	

Components of Physical Exam	
Did I acknowledge vitals and respond to abnormalities with intervention?	☐ N/A ☐ Needs Improvement ☐ Adequate ☐ Good ☐ Mastered
Did I do a full physical exam? Did I fully undress patient, roll patient, unwrap bandages, remove socks?	☐ N/A ☐ Needs Improvement ☐ Adequate ☐ Good ☐ Mastered
Was I able to accurately recognize abnormalities in my physical exam (*crackles at bases, pitting edema, abdominal distention*)?	☐ N/A ☐ Needs Improvement ☐ Adequate ☐ Good ☐ Mastered
Did I respond to abnormalities in physical exam with intervention?	☐ N/A ☐ Needs Improvement ☐ Adequate ☐ Good ☐ Mastered
Did I consciously use my physical exam to support or eliminate differentials I developed during my HPI? Or was I just going through the motion of what I'm supposed to do?	☐ N/A ☐ Needs Improvement ☐ Adequate ☐ Good ☐ Mastered
****THIS IS WHAT I PLAN TO DO DURING MY NEXT CODE TO IMPROVE MY PHYSICAL EXAM:**	

**Components of Generating a Differential**	
Did I begin to generate a broad differential based on EMS report before the patient arrived?	□ N/A □ Needs Improvement □ Adequate □ Good □ Mastered
During the code, was I acknowledging events / vitas / physical exam findings to shape my differential? Or, was I overwhelmed with the distress of the patient / staff and found myself reacting and not thinking?	□ N/A □ Needs Improvement □ Adequate □ Good □ Mastered
Did I properly use my diagnostic tools to eliminate or support my differential? OR, did I "shot gun" general order sets *hoping* to uncover an abnormality I could focus on?	□ N/A □ Needs Improvement □ Adequate □ Good □ Mastered
Did I efficiently use my diagnostic tools (*CXR / Ultrasound / EKG /CT*) to eliminate the most dangerous differential first?	□ N/A □ Needs Improvement □ Adequate □ Good □ Mastered
****THIS IS WHAT I PLAN TO DO DURING MY NEXT CODE TO IMPROVE MY DIFFERENTIALS:**	
**Components of Knowledge Based Tasks-** *examples of knowledge based tasks include intubation, vent settings, STEMI Activations, stroke activations, tPA contraindications,*	
Was I able to recognize what knowledge based tasks were required based on the patient presentation / vitals?	□ N/A □ Needs Improvement □ Adequate □ Good □ Mastered
Was I confident and accurate in pharmacologic doses? (*RSI meds / ACLS meds / pressors / drips / anit-epileptic medications*)	□ N/A □ Needs Improvement □ Adequate □ Good □ Mastered
Was I confident and accurate in bedside scoring? (*stroke scale / wells criteria / Canadian CT rule / SIRS / Sofa*)	□ N/A □ Needs Improvement □ Adequate □ Good □ Mastered
Did I perform well in skilled interventions? (*intubation/ arterial line placement / central line placement*)	□ N/A □ Needs Improvement □ Adequate □ Good □ Mastered
Was I confident and accurate in activating consultant teams? (*stroke team / Cath lab / Ecos team / obstetrics*)	□ N/A □ Needs Improvement □ Adequate □ Good □ Mastered
****THIS IS WHAT I PLAN TO DO DURING MY NEXT CODE TO IMPROVE MY KNOWLEDGE BASED TASKS:**	

**Components of Communication**	
Did I clearly communicate my differentials, thought processes, and goals to the team?	□ N/A □ Needs Improvement □ Adequate □ Good □ Mastered
Did I clearly communicate physical exam findings to the scribe nurse?	□ N/A □ Needs Improvement □ Adequate □ Good □ Mastered
Did I use closed loop communication when important tasks were at stake? (*glucose, lactate, ABGs, medications, interventions, EKGs*)	□ N/A □ Needs Improvement □ Adequate □ Good □ Mastered
Did I frequently summarize progress and communicate concerns to the team and patient?	□ N/A □ Needs Improvement □ Adequate □ Good □ Mastered
****THIS IS WHAT I PLAN TO DO DURING MY NEXT CODE TO IMPROVE MY COMMUNICATION SKILLS:**	

**Components of Leadership**	
Did I effectively establish myself as the team leader?	□ N/A □ Needs Improvement □ Adequate □ Good □ Mastered
Did I maintain the leadership role as interruptions developed or new people arrived to the code?	□ N/A □ Needs Improvement □ Adequate □ Good □ Mastered
Did I effectively manage the tone of the code when things went wrong or the patient decompensated? Did I remain calm?	□ N/A □ Needs Improvement □ Adequate □ Good □ Mastered
Did I maintain proper crowd control? (*med students / EMS / respiratory / techs / family / police*)	□ N/A □ Needs Improvement □ Adequate □ Good □ Mastered
Did I role model professional behavior throughout code?	□ N/A □ Needs Improvement □ Adequate □ Good □ Mastered
****THIS IS WHAT I PLAN TO DO DURING MY NEXT CODE TO IMPROVE MY LEADERSHIP SKILLS:**	

**Components of Teamwork**	
Did I know who the team members were and what was expected of them? Did I know specific names?	□ N/A □ Needs Improvement □ Adequate □ Good □ Mastered
Did I appropriately assign tasks and balance the workload of the team?	□ N/A □ Needs Improvement □ Adequate □ Good □ Mastered
Did I engage team members in decision making? Was I open to ideas from team members?	□ N/A □ Needs Improvement □ Adequate □ Good □ Mastered
Did I foster an overall positive attitude of the team?	□ N/A □ Needs Improvement □ Adequate □ Good □ Mastered
****THIS IS WHAT I PLAN TO DO DURING MY NEXT CODE TO IMPROVE TEAMWORK:**	

**Components of Logistics**	
Did I prepare the room based on the EMS report before the patient arrived? (*set up intubation tools, alert CT a stroke may be on the way, get the ultrasound out for central line*)	□ N/A □ Needs Improvement □ Adequate □ Good □ Mastered
Did I know the names of tools I needed? (*Safe-T-vac suction, oral airway, nasal airway, PEEP-valve, tracheostomy obturator, ect*)	□ N/A □ Needs Improvement □ Adequate □ Good □ Mastered
When I didn't have a tool I needed, did I clearly use support staff? Or alternatively, did I leave the code to find something?	□ N/A □ Needs Improvement □ Adequate □ Good □ Mastered
Did I effectively respond when something wasn't working?	□ N/A □ Needs Improvement □ Adequate □ Good □ Mastered
****THIS IS WHAT I PLAN TO DO DURING MY NEXT CODE TO IMPROVE LOGISTICS:**	

3 things I did extremely well during this code:	***3 things I need to improve on for the next code:***
