# Supplementary material for: Video-assisted self-reflection of resuscitations for resident education and improvement of leadership skills: A pilot study
Source: Perspect Med Educ. 2021 Nov 16;11(2):80–5. doi: 10.1007/s40037-021-00690-9 (PMC8940988; doi:10.1007/s40037-021-00690-9)
Supplement: Supplementary file 2 — 2. Concise Assessment of Leader Management: Previously validated instrument designed by Nadkarni LD, et. al to require minimal user training to assess leadership skills and provide formative feedback. Used by our attending experts to score all 20 videos. [file 40037_2021_690_MOESM2_ESM.pdf]

Intended for 2 objective attendings to evaluate resident leader of the medical resuscitation. This will be completed for both groups (*residents who will view video feedback, and residents who will not*) before and after video analysis intervention. The goal will be to compare the delta in CALM scores between the residents who get the video analysis intervention and the residents who reflect without video. Tool replicated from Dr. Kessler. Designed for minimal user training and real-time assessment of data. Has shown high inter-rater reliability.

Concise Assessment of Leader Management*

Leadership

Announced role as leader	☐ Yes ☐ No
Clear Role as leader throughout case	☐ Rarely ☐ Sometime ☐ Mostly ☐ Always
Style appropriate and effective for situation	☐ Rarely ☐ Sometime ☐ Mostly ☐ Always

Communication

Voice Appropriately loud and clear	☐ Rarely ☐ Sometime ☐ Mostly ☐ Always
Addresses people explicitly (*by name*)	☐ Rarely ☐ Sometime ☐ Mostly ☐ Always
Reinforces closed loop communication	☐ Rarely ☐ Sometime ☐ Mostly ☐ Always

Team Management

Assigns or Acknowledges Roles	☐ Rarely ☐ Sometime ☐ Mostly ☐ Always
Directs team effectively / assigns tasks	☐ Rarely ☐ Sometime ☐ Mostly ☐ Always
Balances work load of team	☐ Rarely ☐ Sometime ☐ Mostly ☐ Always
Engages team members in decision making	☐ Rarely ☐ Sometime ☐ Mostly ☐ Always
Summarizes case status periodically	☐ Rarely ☐ Sometime ☐ Mostly ☐ Always

Medical Management

Prioritizes task order	☐ Rarely ☐ Sometime ☐ Mostly ☐ Always
Maintains global view (*avoids fixation bias*)	☐ Rarely ☐ Sometime ☐ Mostly ☐ Always
Periodically reassesses patient	☐ Rarely ☐ Sometime ☐ Mostly ☐ Always
States next steps in patient care	☐ Rarely ☐ Sometime ☐ Mostly ☐ Always
Aware of limitations and seeks help as needed	☐ Rarely ☐ Sometime ☐ Mostly ☐ Always

*Replicated from:

Nadkarni LD, Roskind CG, Auerbach MA, Calhoun AW, Adler MD, Kessler DO. The Development and Validation of a Concise Instrument for Formative Assessment of Team Leader Performance During Simulated Pediatric Resuscitations. Society for Simulation in Healthcare. 2017.
